# Applying the Model for Assessing the Value of AI (MAS-AI) Framework To Organizational AI: A Case Study of Surgical Scheduling Assessment in Italy

**DOI:** 10.1007/s10916-025-02235-7

**Published:** 2025-08-23

**Authors:** Valentina Bellini, Francesco Calabrò, Elena Bignami, Tudor Mihai Haja, Iben Fasterholdt, Benjamin SB Rasmussen, Rossana Cecchi

**Affiliations:** 1 Anesthesiology, Critical Care and Pain Medicine Division, Department of Medicine and Surgery, University of Parma, Viale Gramsci 14, Parma, 43126 Italy; 2 Laboratory of Forensic Medicine, Department of Medicine and Surgery, University of Parma, Parma, Italy; 3 CIMT - Centre for Innovative Medical Technology, Odense University Hospital, Odense, Denmark; 4 Program for Health System and Technology Evaluation, Toronto General Hospital Research Institute, University Health Network, Toronto, Canada; 5 Department of Radiology & CAI-X - Centre for Clinical Artificial Intelligence, Odense University Hospital, Odense, Denmark

**Keywords:** Artificial intelligence, Machine learning, Assessment, HTA, Liability, Legal medicine

## Abstract

This work aims to explore the transferability of the *Model for Assessing the value of Artificial Intelligence in medical imaging* (MAS-AI) in the Italian context through a case-study.

We applied the MAS-AI, a model for assessing AI in healthcare, to fulfil a technology assessment of an AI model developed within *our institution.* The model, called *New organization model for the surgical unit* (BLOC-OP), uses AI to improve the schedule efficiency of the surgical unit. The analysis of BLOC-OP’s features, as they were described in the project presentation, was conducted through the requirements for the assessment contained in the MAS-AI model.

The methodological framework of MAS-AI was fully followed, allowing us to conduct a comprehensive assessment of the BLOC-OP model in all its aspects. We provided a detailed description of each domain within the framework, along with a summary table.

The case study demonstrates the feasibility of applying MAS-AI to organizational AI models in a national context different from where the framework was originally developed. Rather than proposing a new model, we tested the adaptability of MAS-AI in evaluating a non-imaging AI system. This confirms its flexibility beyond its original scope and supports its potential as a generalizable tool for AI evaluation in healthcare.

## Introduction

Artificial intelligence (AI) is increasingly being integrated into healthcare systems, where it promises to improve efficiency, accuracy, and personalization of care. AI models, particularly those based on machine learning and deep learning, can detect complex patterns within clinical data, facilitate early diagnosis, optimize therapeutic choices, and support real-time decision-making processes. These technologies have the potential to address longstanding inefficiencies in healthcare delivery and enable a shift from reactive to proactive and predictive models of care [1, 2]. Moreover, the surge in digital health records and biomedical imaging has created an ideal substrate for AI development, accelerating its deployment across clinical and administrative domains.

AI is capable of rapidly analysing vast amounts of data and clinical documentation and it can be very useful for numerous decision-making activities in medicine, such as diagnosis, therapy, prognosis and patient monitoring management [3]. The scientific literature has highlighted numerous contexts in which AI has been applied, including radiology, radiotherapy, ophthalmology, dermatology, gastroenterology, gynaecological oncology, haematology and infectious diseases, including the diagnosis of COVID-19 [4, 5].

However, the regulation of AI products in medicine is, to date, still rather patchy. Existing processes, such as Health Technology Assessment (HTA) [6] and European Commission (CE) marking [7], are not able to fully frame the specificities of AI-based models and they do not represent the expected guarantee of quality and robustness [8]. A systematic review conducted in 2021 found that almost all articles in the literature agree that AI is an exception to HTA processes [9]. In fact, most authors consider current HTA processes as outdated when applied to AI and emphasise the importance of innovation in this area [10]. Therefore, an innovation or improvement in regulatory methodologies should be necessary.

In this context, both regulatory authorities and academic scholars have increasingly called for updated, flexible frameworks that can address the adaptive, non-deterministic, and often opaque nature of AI technologies in healthcare. For example, the European Commission published in 2021 a Proposal for a Regulation on Artificial Intelligence, known as the *Artificial Intelligence Act*, which introduces a risk-based regulatory approach for AI systems, including specific obligations for those used in medical contexts [11]. The Act defines “high-risk” systems and requires transparency, human oversight, and robustness testing, but its implementation is still ongoing and subject to further revisions. Similarly, in the United States, the Food and Drug Administration (FDA) issued an Action Plan for AI/ML-Based Software as a Medical Device (SaMD), which embraces a total product lifecycle approach and aims to incorporate real-world performance monitoring and iterative improvement while ensuring patient safety [12]. Ethical and legal scholars have also emphasized the necessity for explainability, accountability, and fairness in AI healthcare applications, particularly when these systems influence clinical decision-making or patient outcomes [13, 14]. Without adequate governance mechanisms, AI deployment in medicine could amplify biases, generate opaque risks, or weaken professional liability structures.

The aim of this work is to explore the potential of an expanded HTA model and evaluate its transferability into a context different from the one in which it was developed. Specifically, we applied the MAS-AI framework, originally designed for medical imaging technologies, to a novel AI system developed for the optimisation of surgical block management (BLOC-OP), within an Italian healthcare facility. After a systematic application of the nine domains and five process factors of the MAS-AI model, the study aims to assess its feasibility, adaptability, and suitability for evaluating organisational and workflow-oriented AI solutions.

## Methods

### The evaluating model – “MAS-AI”

The Model for Assessing Artificial Intelligence (MAS-AI) [15] was proposed at the end of 2022 with the aim of addressing the shortcomings of traditional HTA methodologies in assessing AI technologies, specifically the ones applied to medical imaging.

The model was developed by a multidisciplinary team. Professionals from different fields, including HTA experts, were involved, including health economists, clinicians, radiologists with technical, legal, ethical and patient expertise.

**Fig. 1:**
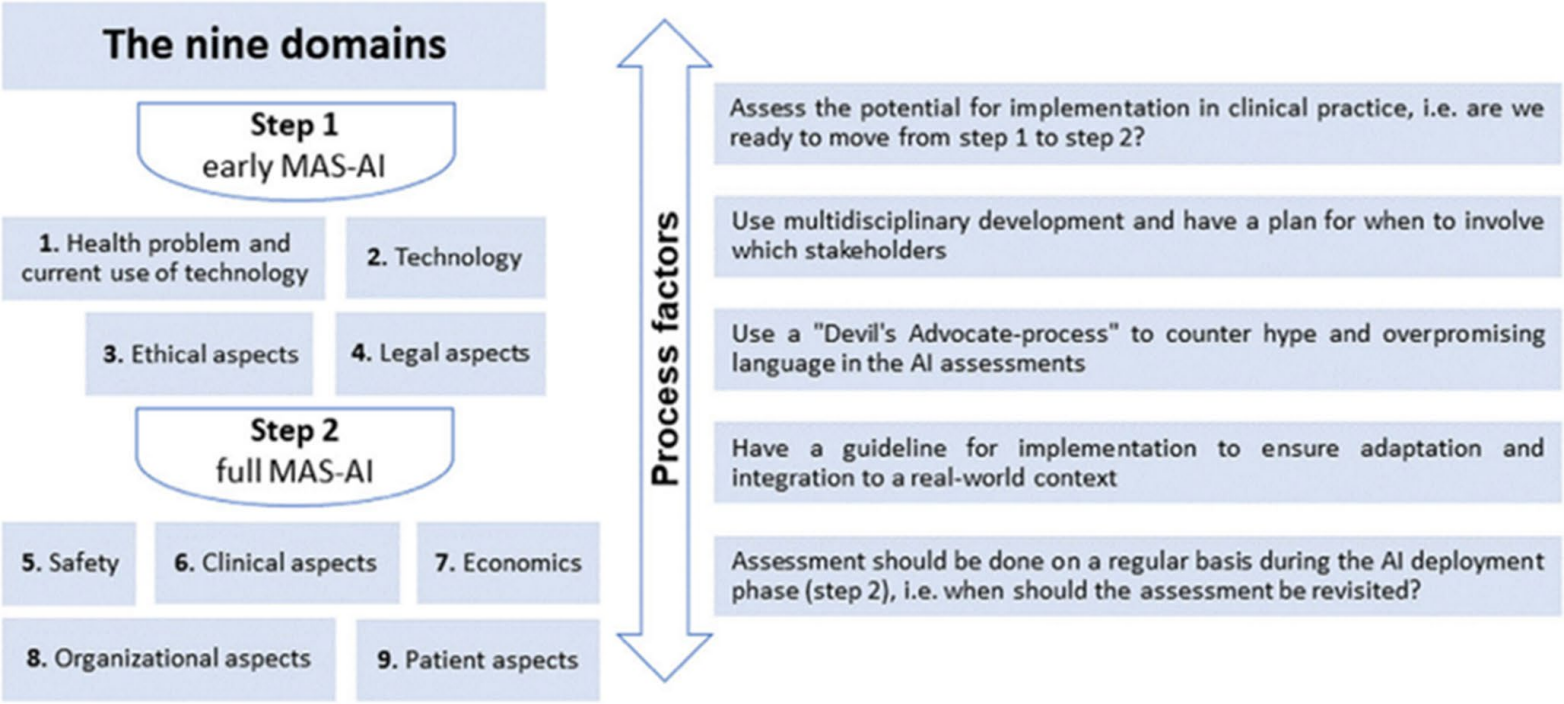
The nine domains of the MAS-AI model and next to them five so-called “process factors”. (reprinted with permission from Fasterholdt et al. (ref nr. 15) - © The Author(s), 2022)

The MAS-AI model consists of three parts and Fig. 1 provides an overview of the contents of these parts. There are two steps covering nine domains and five process factors for a MAS-AI evaluation. The authors suggest that the order of the domains is of no particular significance. Step 1 contains a description of the patients, how the AI model was developed and initial ethical and legal considerations.

Completing the four domains of phase one is a prerequisite for moving on to phase two. In phase two, a multidisciplinary evaluation of the results of the AI application is carried out for the five remaining domains: safety, clinical aspects, economic aspects, organisational aspects and patient aspects.

The last part consists of five process factors to facilitate a good evaluation of the process by which the AI system was developed.

In the chapter ‘*Transferability and Perspectives*’ of the article on MAS-AI, the authors reiterate how their model can be sufficiently generic to also evaluate other types of AI in healthcare, in addition to imaging products [15].

Following this indication, in this work we verified the applicability of the method to the ‘BLOC-OP’ model. The intention is to preliminarily explore the transferability of an AI model validation in the Italian context.

### The technology to be evaluated - “BLOC-OP”

BLOC-OP is an AI model that was developed at the *2nd Operational Unit of Anaesthesia and Intensive Care*, within the General and Specialist Surgical Department of the *Azienda Ospedaliero-Universitaria* of Parma in Italy.

The main aim of this project is the creation of an integrated technological-organisational model capable of exploiting data from the operating theatre to optimise the management and organisation of the operating block (Table 1). In particular, the model is intended for scheduling surgical operations through the integration of clinical and anamnestic information, as well as data relating to surgical timings and post-operative hospitalisation times. The goal is to optimise the organisation of the surgical compartment in order to improve the efficiency and quality of care [16]. In addition to the primary objective of creating a scheduling technology for surgical interventions, the project also includes an analysis of the possible impact of this organisational system on the economy of the operating block, patient waiting times and the number of unscheduled admissions to intensive care. Finally, the evaluation will also focus on comparing the performance of the operating block organisation, e.g. considering the occupancy times of operating theatres, operating session times and the activation rate of the medical team, with the performance obtained by the new scheduling system [16].

**Table 1:**
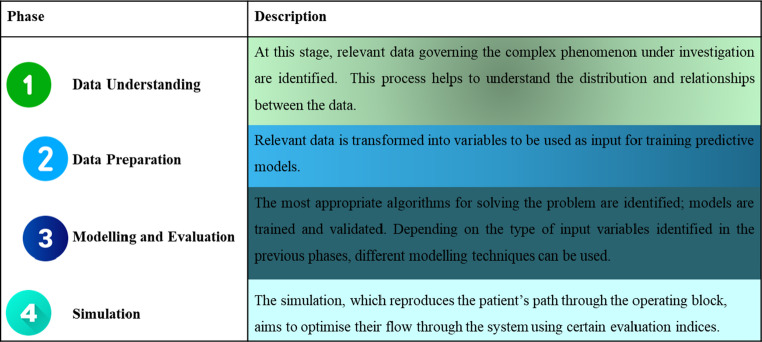
The structure of the project phases of BLOC-OP technology

A comparison between the data obtained through the traceability system and the information system in use, called Ormaweb [17] (or *O4C*), is planned in order to assess the effectiveness of the new system in providing accurate and reliable data. Secondary endpoints include the recording and analysis of data from the post-operative recovery room, the comparison of the performance of the new scheduling system with the one currently in place, based on estimates of surgical and recovery times in the post-operative recovery room, the comparison of data from Ormaweb system with those obtained through the new tracking system, and the evaluation of number of total admissions to the intensive care unit. The BLOC-OP project is not yet deployed for clinical use.

In the next section we analyse the protocol of the BLOC-OP project, according to the 9 domains of the MAS-AI model [15].

## Results

**Fig. 2:**
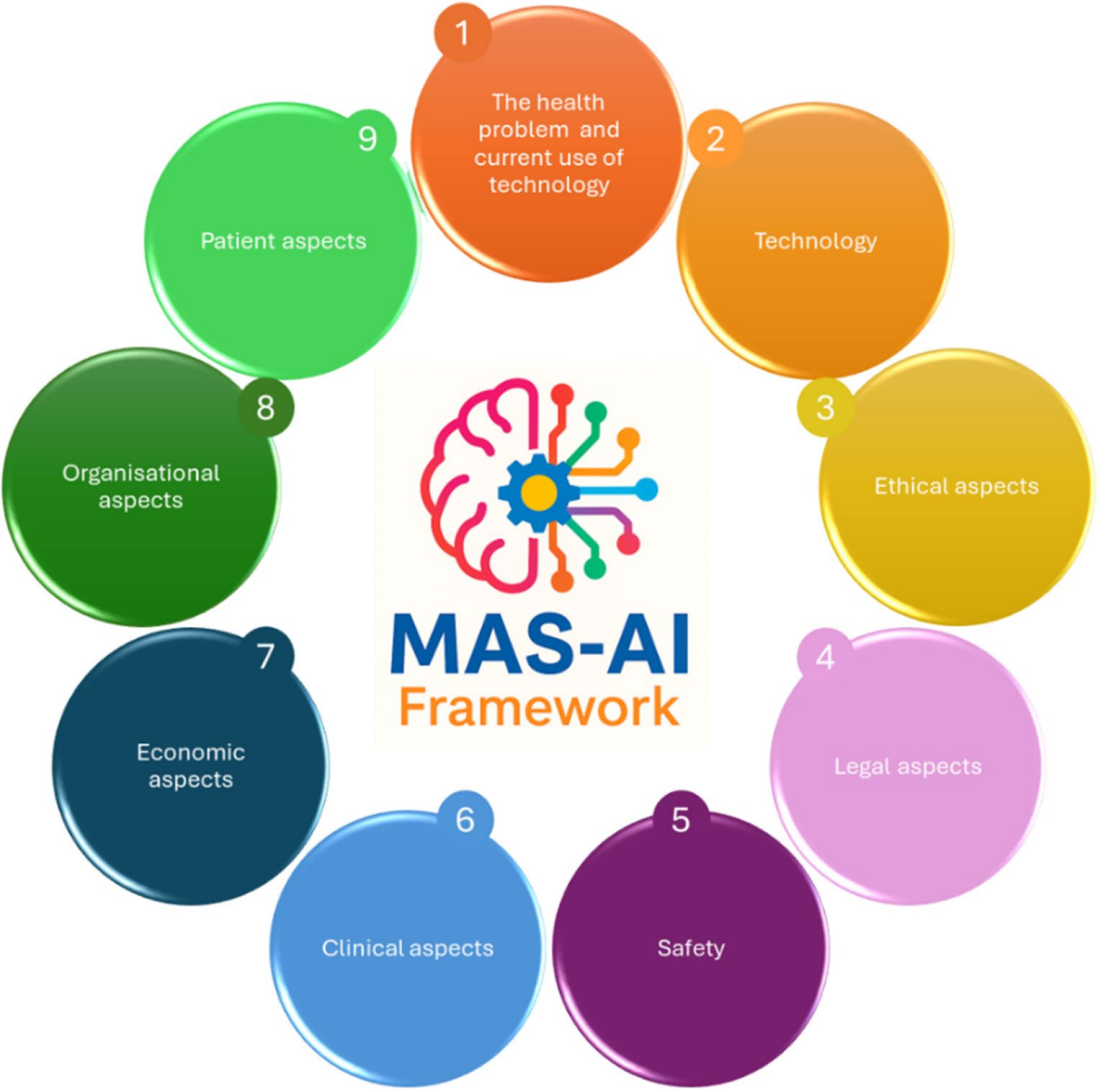
Graphical representation of the MAS-AI framework, illustrating its nine domains [15]

We applied the 9 domains of MAS-AI, illustrated in Fig. 2, to the BLOC-OP technology, fulfilling a full MAS-AI assessment comprehensive of: the detailed description of each domain, the answers to the 5 “process factors”, and, finally, a summarizing table of the assessment.

### Domain 1: The health problem and current use of technology

The health problem related to operating block management involves a significant margin of error due to planning based on operators’ personal experience. Procedures in the hospital involve weekly meetings between surgeons and anaesthesiologists to validate scheduling, mostly based on the average duration of surgery, but this can lead to improper use of operating rooms [18]. To address this problem, the use of artificial intelligence and machine learning techniques is emerging as a promising solution. Recent studies have shown that the application of machine learning models can improve the scheduling accuracy of surgical operations and reduce delays, thereby optimising resource allocation [19].

However, the effective application of such models requires data of high quality and accuracy. Today, in Parma’s University Hospital, the recording of patient times and movements in the operating block is done manually, via a information system called “Ormaweb”. The use of an automated patient tracking system could improve data quality and reduce human errors. An autonomous registration system could also lighten the workload of the operators and reduce the changeover time between patients. A positive example is the experience of the hospital in Cesena, an Italian city, where the implementation of aRadio Frequency Identification (RFID)- based tracking system has improved the accuracy and completeness of trauma patient documentation [20]. We expect that the application of AI could elaborate all the data coming from an automated patient tracking system, while taking into account all the variables that affect the succession of surgeries. This could avoid the errors of manual planning in the surgery and lead to more effective clinical and organisational management of the operating theatre.

### Domain 2: Technology

The authors of the MAS-AI argue that the technological aspects of the evaluation can be divided into two main categories: those related to the development of the technology and those related to its implementation in the specific clinical context being evaluated. In this case, the BLOC-OP protocol is mainly concerned with the former, so our focus will be more on evaluating those. Furthermore, Domain 2 of the MAS-AI refers to the CLAIM (Checklist for AI in Medical Imaging) [17] guidelines, which are not applicable in this case because BLOC-OP is an AI system but is not based on imaging. Therefore, we will limit ourselves to framing the technological aspects according to the description provided in the BLOC-OP’s project protocol. In terms of technology development, the BLOC-OP project consists of two phases. The first phase consists of the systematic collection of data from the central operating block, the Recovery Room and the on-site monitoring of vital parameters. During this phase, data will be collected on operating room times, patient biographical and anamnestic data, data from the Recovery Room and data on hospital stay.

In order to ensure the traceability of patients, a system consisting of a Bluetooth tag associated with the patient’s identification code will be used, which will be placed on the medical record when entering the operating theatre. The operating theatres and the Recovery Room will be equipped with Raspberry Pi systems capable of detecting and storing the movements of the tag within the wards. The data recorded by the Raspberry Pi systems will be periodically extracted via Bluetooth technology using dedicated tablets. The second phase of the project involves the application of calculation techniques to develop a technology capable of providing an optimised organisation tool for the operating block. During this phase, Machine Learning techniques may be used to solve complex problems where it is not possible to define specific instructions or rules to achieve an optimal solution. The BLOC-OP is based on the analysis of a large dataset, which is used to build a predictive analysis system. To enable the discovery of hidden patterns, a large number of data must be analysed. In this case, approximately 50 variables were identified for each patient, which will constitute a single instance of the dataset.

The size of the dataset is compatible with a 6-month data collection campaign for a total of 1,200 patients. The dataset was divided into three parts:

1. **Training set** (60%): This data set is used to train the algorithm and build the model. During this phase, the algorithm learned the correlations in the data.
2. **Validation set** (20%): This validation set is used to calculate the accuracy or error of the classifier and to remodel parameters based on the results obtained. Instances of the validation set are not used during model creation or optimisation.
3. **Test set** (20%): This data set represents the future data that will be analysed. It is used in a similar way to the validation set but was never used during the creation or optimisation of the model.

The development of the BLOC-OP AI technology follows three basic steps:

1. **Data understanding**: During this phase, relevant data are identified to create specific models governing the different parts of the complex phenomenon being examined. A univariate analysis is performed to examine the characteristics of each variable in the data set. This process helps to better understand the distribution and relationships in the data.
2. **Data preparation**: In this phase, data identified as relevant are processed using feature engineering. Feature engineering consists of transforming the data into other variables suitable for use as input in predictive models. During this phase, data normalisation, a process that rescales the variables so that they are comparable on the same scale, preventing some variables from having too much weight compared to others, may be required.
3. **Modelling and Evaluation**: In this phase, the most suitable algorithms for the problem are identified and the training and validation of the model created by the selected algorithm is carried out. Depending on the input variables identified in the previous phase, different modelling techniques can be used. For example, if the relationships between the data are clear, a Bayes classifier or a Decision Tree, which are algorithms based on probability calculation, could be used. Conversely, if the relationships are more complex, neural networks and deep learning can be used for modelling.

Although the BLOC-OP system is not imaging-based and the CLAIM guidelines were developed specifically for medical imaging AI tools, several relevant principles from CLAIM can be extrapolated [21]. In particular, the need for reproducibility, technical transparency, and performance validation are highly pertinent. During development, one of the main challenges was integrating heterogeneous data sources—including surgical schedules, preoperative information, and intraoperative timings, into a unified and structured dataset. Ensuring data quality required custom pipelines for data cleaning, synchronization, and validation [10].

Another technical hurdle involved the traceability and reliability of Bluetooth-based patient tracking, which had to be tested under variable physical conditions within the operating block. Close collaboration with clinical engineers was essential to ensure compatibility with existing hospital IT infrastructure and to avoid interference with Ormaweb/O4C systems. Furthermore, ensuring compliance with data protection regulations such as the GDPR demanded early anonymization strategies and strict data access protocols [22]. Although the project predated specific AI ethical frameworks, an emphasis was placed on human oversight, data security, and explainability throughout the design.

### Domain 3: Ethical aspects

The project team of the BLOC-OP project assure that the study was conducted in accordance with Good Clinical Practice (GCP) standards and the Declaration of Helsinki [23]. Both documents represent an international standard of ethics and quality required for the design, conduct, recording and reporting of clinical trials involving human subjects. Compliance with them ensures compliance with basic ethical principles such as the protection of the rights, safety and well-being of research participants, and fairness in the assessment of risks and benefits. However, the authors do not explicitly refer to ethical aspects that specifically concern AI systems.

In fact, current AI-based clinical decision support systems (CDSSs) raise significant ethical challenges that extend beyond traditional research ethics frameworks. These include the delegation of decision-making processes to non-transparent, “black box” algorithms, which can obscure responsibility attribution in the event of diagnostic or organisational errors, and compromise clinicians’ autonomy and accountability. The lack of explainability also poses a barrier to trust, informed consent, and contestability of automated outputs. As highlighted by Bertl et al. (2023), these unresolved issues remain among the main barriers to the clinical adoption of AI technologies in healthcare decision-making processes [24, 25].

As a guarantee of transparency, the project allows the Ethics Committee to monitor, verify, review and inspect data and original documents, in order to ensure compliance with ethical standards and regulations. However, it should be noted that the BLOC-OP project was conceived before the publication of specific ethical guidelines on the development and application of AI in healthcare.

### Domain 4: Legal aspects

During the development of BLOC-OP lawyers with experience in the AI regulation field were consulted to ensure compliance with the regulatory framework and to adapt the informed consent forms. Patients were in fact invited to participate in the study through informed consent, and only included once consent has been obtained. Furthermore, the BLOC-OP, being an organisational technology, is not considered a medical device. Therefore, the CE marking, with its associated procedures, does not apply in this case [7].

### Domain 5: Safety

The BLOC-OP implements several measures to guarantee the security of the system. Access to the local network, which is used for communication between devices, requires authentication using Wi-Fi Protected Access 2 (WP2) encryption. WP2 encryption is a security standard that uses advanced cryptographic algorithms to protect the Wi-Fi connection from unauthorised access and passive or active attacks. In addition, access to the local network is subject to MAC address constraints. The MAC address is a unique identifier assigned to each network device. The use of MAC address constraints makes it possible to limit network access to authorised devices only, preventing access by unauthorised or unrecognised devices.

The storage system adopted by the BLOC-OP is based on the non-relational database MongoDB. MongoDB is a NoSQL database that offers a flexible structure for organising data and provides advanced security features. This includes the ability to encrypt data access, ensuring that only authorised users can access sensitive information. To ensure data reliability and availability, the system uses Redundant Array of Independent Disks (RAID) media. RAID is a technology that combines multiple hard disks into a single logical drive, providing redundancy and reducing the risk of data loss. RAID media allow data to be replicated and distributed across multiple disks, ensuring continuity of operations even in the event of hardware failure or read/write errors. The authors also point out that the components of the medical record traceability system are not considered medical devices according to the *Italian Legislative Decree 46/1997* [26]. Furthermore, these components will not interact with the computer systems already in use at the hospital. Compatibility with existing systems has been confirmed by the Clinical Engineering Departmental Structure.

### Domain 6: Clinical aspects

While it can be inferred that there will be important positive effects for patients secondary to improved intervention planning, as it is an organisational technology, it is more difficult to determine the exact clinical effects (e.g. in terms of effects on mortality, morbidity, QoL, etc.). The authors hope to be able to measure these factors at a later stage, after the actual implementation of the scheduling system with AI.

### Domain 7: Economic aspects

The authors claim, as one of the secondary objectives of the study, that an analysis of the impact of the system in terms of the economy of the operating block will be carried out. It is hypothesised that a more efficient organisational system that allows an increase in the number of operations performed can also have positive effects on hospital economics. One of the secondary objectives is an analysis of the impact of this organisational system on the economy of the operating block, considering both the negative impact of the costs of developing and implementing the system, and the positive impact of the savings secondary to the efficiency of the scheduling.

### Domain 8: Organisational aspects

After the training of the AI algorithm, the BLOC-OP workflow will be able to process the variables necessary to draw up an appropriate operating block schedule, starting with the basic logic concerning the presence of doctors on shifts and the availability of operating theatres. Once the waiting list of patients has been pre-arranged, the system will use this information to define the planning of interventions, also ensuring a correct order according to the priority codes assigned to individual patients. The ultimate goal is to create a technology that, taking into account all the relevant information for this decision-making process, styles the most effective planning possible, in a completely autonomous manner. In addition, multidisciplinary work is planned with a team of management engineers to arrive at improvement proposals in a strictly organisational sense.

### Domain 9: Patient aspects

In the context of the BLOC-OP, the risk/benefit assessment ensures that patients are not exposed to additional risks compared to standard treatment. The technology, at this stage, is simply based on monitoring the timing of surgical operations, without changing the way patients are treated or their time on the waiting list. There is therefore neither a direct benefit nor an additional risk at the technology development stage. The benefits could emerge in the event that actual deployment reduces waiting times and offers better access to care.

In addition, a collection of reports from patients involved in the study is planned to collect any feedback from them. There is currently a client interface, hosted by the central server, that offers several functionalities. Patient enrolment is done by a member of the medical staff when the patient enters the operating block to undergo surgery. The enrolment is performed using a tablet device that communicates with the web application and by providing the necessary information. The system also provides a graphical interface able to monitor in real time the various movements of patients.

### Process factors for a MAS-AI assessment

The so-called ‘process factors’ of MAS-AI assessment were submitted to the project team of the BLOC-OP in order to obtain a self-assessment of certain aspects of project development (Table 2). We report directly the answers received.

**Table 2:** The assessment of the process factors^1^

1. Regarding the maturity of the model, is it still in the retrospective development phase or does it already have approval for implementation and prospective evaluation?	“Yes, it has already been approved with the Ethical Committee and AOUPR (Azienda Ospedaliero-Universitaria di Parma) but is not yet deployed for clinical use”
2. Did you use a multidisciplinary approach involving experts from different fields?	*“Yes*,* we work with a multidisciplinary team composed not only of Anaesthesia and Intensive Care specialists*,* but also of software*,* clinical and management engineers.”*
3. Did you use a so-called ‘devil’s advocate’ attitude to counterbalance the hype and excessive optimism, e.g. by including people in the team who were sceptical about AI applications?	*“Yes*,* we have been working with both Unipr and AOUPR engineers. In the project group there are both those who obviously believe in AI and those who were potentially sceptical and decided to join once they got to know the project better.”*
4. Have you already prepared guidelines for the actual implementation of the model?	*“Not yet*,* but we will try to follow an implementation”*
5. Assessment should be done on a regular basis during the AI deployment phase, so when should the assessment be revisited?	*“At date*,* we regularly check the IoT equipment. If it comes to implementation*,* there will certainly be regular checks of the whole system.”*

^1^ the MAS-AI model recommends a broader update; not just at check of the AI-models performance or the technical aspects, but to revisit the entire evaluation

### Summary table of the assessment

The MAS-AI model suggests that at the end of a full assessment a summary table should be produced (Table 3).

**Table 3:** Structured analysis of the BLOC-OP project according to the MAS-AI model criteria

STEP 1 – early MAS-AI		
	Domain Description	BLOC-OP analysis
1	The health problem and current use of technology	The management of operating block is very complex and is currently imprecise and based on a very limited number of variables. Initial results in the literature regarding the use of AI for this purpose were promising. Therefore, it was decided to test the use of AI models to improve surgical scheduling and simultaneously exploit an indoor tracking system to optimize the data quality and reliability.
2	Technology	The first phase consisted of the collection of a solid dataset, also exploiting indoor tracking system for the direct extrapolation of surgical and recovery room times. The second phase was the application of AI techniques to be able to automatically process the data and provide models for predicting operating room and recovery room occupancy and then scheduling surgical procedures.
3	Ethical aspects	The study was conducted in accordance with Good Clinical Practice (GCP) standards and the Declaration of Helsinki.
4	Legal aspects	The authors consulted with a team of experienced AI lawyers to ensure compliance with the regulatory framework and to adapt the informed consent forms.
STEP 2 – full MAS-AI		
5	Safety	Security measures were designed together with software engineers. BLOC-OP technology adopts several security measures such as WP2 encryption, Media Access Control (MAC) address constraints, the use of MongoDB databases and RAID media to guarantee data protection, limit access only to authorised devices and ensure the reliability and availability of information.
6	Clinical aspects	The clinical aspects are mainly related to the fact that a better optimisation of resources leads to an increase in the quality and safety of the services provided.
7	Economic aspects	The economic impact of the new system is among the secondary objectives of the project and to be investigated later on. More accurate scheduling is expected to lead to an optimisation of available resources.
8	Organisational aspects	The ultimate goal is to create a technology that takes into account all the information relevant to this decision-making process, and that is autonomous in making the most effective plan. In addition, the model will be designed to be easily implemented in daily clinical practice.
9	Patient aspects	The technology does not place patients at any additional risk. Moreover, in the process of collecting informed consent, patients are given information about the use of AI techniques.

## Discussion

As well known, prevention of critical issues that can arise applying AI systems in medical field is of paramount importance [27]. MAS-AI model helps in identifying criticalities in AI systems.

The case study, presented as a practical example of the application of the MAS-AI guideline to an Italian research project involving AI, proved the technology to be overall coherent to its purposes. All domains could be filled, and the structure of BLOC-OP project’s presentation matched the requirements for the MAS-AI assessment. Those requirements are therefore in line with what is required within a project protocol in Italy. The division into 9 domains proposed in the MAS-AI makes the presentation clearer and allows for a quicker orientation of the specific aspects covered.

Fundamental was the possibility of applying the MAS-AI from an ethical and legislative point of view also in Italy, a context that is culturally significantly different from the Danish one in which MAS-AI was developed. This opens up the prospect of being able to devise an AI assessment method on a global level through adjustments that we have seen to be possible.

Since MAS-AI was published in 2022, the validity and transferability of MAS-AI has been thoroughly investigated in a Delphi study lead by Iben Fasterholdt (this study is currently under review). The model’s generalizability is also investigated in cancer screening in the joint action project EUCanScreen [28] and in digital technologies including AI in the horizon project EDiHTA [29]. Thus, we are currently updating the MAS-AI tool to make it even more applicable to areas outside medical imaging. However, already most components of the MAS-AI are transferable components relevant to broader AI evaluation according to a recent overview of AI evaluation frameworks [30] and also the unpublished results from the Delphi study support this claim.

As assumed by the authors of the MAS-AI, the least transferable domain is domain 2 because it is linked to the CLAIM guidelines, thus appliable only to the field of imaging. The BLOC-OP protocol precisely describes the technological aspects; however, these could be supplemented with references to guidelines or best practices relating in this case to the management domain. The MAS-AI through the final section of the five so-called *‘process factors*’ allows a more comprehensive evaluation of the project than the protocol alone. These questions in fact provide an overview of how the authors of BLOC-OP are working in developing their technology.

### Limitations

This MAS-AI evaluation of the BLOC-OP project presents several limitations. First, the assessment is based exclusively on the project protocol, as the system has not yet been deployed in clinical practice. Accordingly, there is limited availability of hard data across several domains, particularly safety, clinical, economic, organisational, and patient aspects, which had to be assessed theoretically. This implies that the level of evidence remains low, as the protocol reflects the project group’s intentions rather than measurable outcomes. Second, the evaluation was conducted within a single healthcare institution, using a specific organisational and technological infrastructure. As such, the generalisability of the findings to other contexts, whether institutional, regulatory, or technological, may be limited. Broader external validation will be essential to confirm the applicability and robustness of the MAS-AI framework in diverse environments. Third, some domains within the MAS-AI framework, especially Domain 2, originally inspired by the CLAIM checklist and tailored to imaging technologies, required interpretative adaptation to fit an organisational AI model. This process, while feasible, highlights the need for further refinements to increase the framework’s specificity and relevance for non-imaging, system-level AI applications. Fourth, the evaluation did not include a formal comparison with alternative AI assessment or health technology assessment (HTA) frameworks. While MAS-AI demonstrated applicability and comprehensiveness, a critical appraisal of existing methodologies could have provided a broader context and potentially strengthened the overall analysis. Finally, some elements of the assessment remain incomplete or preliminary due to the lack of implementation data. Nevertheless, MAS-AI is designed as an iterative and updatable framework. In line with the fifth process factor that emphasises the need for continuous improvement, additional real-world data will be integrated as the BLOC-OP system progresses through implementation and prospective evaluation.

## Conclusions

This case study demonstrates that the MAS-AI model, originally developed for the evaluation of AI in medical imaging, can be effectively applied to a different technological and national context. By systematically assessing the BLOC-OP project, a non-imaging AI system for surgical scheduling, across the nine domains and five process factors of MAS-AI, we confirmed the framework’s comprehensiveness, flexibility, and methodological clarity. The analysis supports MAS-AI’s potential as a standardized tool for AI evaluation in diverse healthcare settings, including organizational and workflow-related innovations.

Future research will focus on the clinical implementation of the BLOC-OP model within the operating block of our institution. This will enable the collection of prospective performance data on operating room utilization, patient flow, and scheduling accuracy. Additionally, further studies will investigate the usability and acceptance of the system by healthcare professionals, and assess its impact on patient outcomes. At a methodological level, future work should explore how MAS-AI can be aligned with evolving international regulatory frameworks, and whether domain-specific extensions (e.g., for organizational AI) could enhance its applicability. Cross-institutional validation studies are also warranted to test the model’s generalizability across different healthcare environments.

## Data Availability

No datasets were generated or analysed during the current study.
